# Dietary niche partitioning of three Sky Island *Sceloporus* lizards as revealed through DNA metabarcoding

**DOI:** 10.1002/ece3.10461

**Published:** 2023-09-07

**Authors:** Erin P. Westeen, José G. Martínez‐Fonseca, Christian A. d'Orgeix, Faith M. Walker, Daniel E. Sanchez, Ian J. Wang

**Affiliations:** ^1^ Department of Environmental Science, Policy, and Management University of California, Berkeley Berkeley California USA; ^2^ Museum of Vertebrate Zoology University of California Berkeley Berkeley California USA; ^3^ School of Forestry Northern Arizona University Flagstaff Arizona USA; ^4^ Department of Biology Virginia State University Petersburg Virginia USA; ^5^ Pathogen and Microbiome Institute Northern Arizona University Flagstaff Arizona USA

**Keywords:** biotic interactions, competition, resource use, species coexistence, spiny lizards

## Abstract

Lizard diets are highly diverse and have contributed to the diversification, biogeographical distributions, and evolution of novel traits across this global radiation. Many parts of a lizard's ecology—including habitat preferences, foraging modes, predation risks, interspecific competition, and thermal constraints, among others—interact to shape diets, and dietary niche partitioning simultaneously contributes to co‐occurrence within communities. We used DNA metabarcoding of fecal samples to identify prey items in the diets of three sympatric *Sceloporus* lizards in the Madrean Sky Islands of Arizona, USA. We found evidence for dietary niche partitioning between interacting species concomitant with their respective ecologies. We also compared diet composition between populations to understand how conserved or plastic species' diets are between different environments. Our findings suggest that habitat generalists are also diet generalists in this system, while the same may be true for specialists. The identification of prey items to much lower taxonomic levels than previously documented further reveals hidden diversity in the diets of these species and underscores the utility of metabarcoding for understanding the full complexity of lizard diets.

## Graphical abstract

We used DNA metabarcoding to quantify dietary breadth and overlap for three species of Sceloporus lizards in the Madrean Sky Islands, Arizona, USA. We uncovered evidence for dietary niche partitioning between interacting species, which may facilitate their ability to co‐occur. We present previously unreported diet items for each of the three lizard predator species.

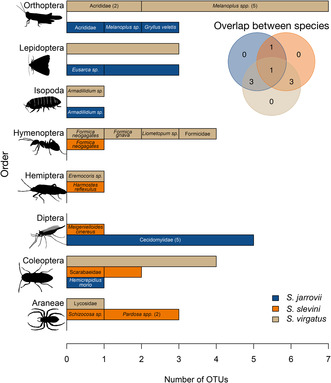

## 1 INTRODUCTION

Feeding ecology is one of the most essential aspects of an organism's life and plays a key role in the evolution of biodiversity. Incredible diversity in diet, levels of dietary specialization, and feeding modes and strategies have evolved across the tree of life (Fryer & Iles, 1972; Lovette et al., 2002; Schluter, 1993). Diet evolution is governed simultaneously by ecological opportunity and competition (Schluter, 2000), and dietary divergence represents an important form of resource partitioning that can enable species co‐occurrence (Ford et al., 2016; Kartzinel et al., 2015; Pianka, 1973; Schoener, 1974). In squamate reptiles, dietary shifts are sufficiently important to influence diversification (Grundler & Rabosky, 2021; Vitt & Pianka, 2005), structure assemblages (Losos, 1994; Vitt & Pianka, 2005), and promote the evolution of novel morphologies (Savitzky, 1981; Vitt & Zani, 1996).

Other aspects of an organism's ecology and behavior intersect with patterns of prey consumption. Microhabitat preferences or requirements, thermal constraints, competitive interactions, and predation risks can all influence spatial and temporal foraging opportunities (Gordon et al., 2010; Lopez‐darias et al., 2012; Novosolov et al., 2018; Svanbäck & Bolnick, 2007). Because prey species are not evenly distributed across landscapes, these factors influence the diet items available to predator species. Dietary niche partitioning within communities is often a result of these many interacting elements and can enable sympatry by reducing competitive overlap (Pianka, 1973; Schoener, 1974).

Many studies focus on how interspecific diet differs within communities (Pacala & Roughgarden, 1985; Serrano‐Cardozo et al., 2008; Vitt & de Carvalho, 1995), but fewer examine the consistency of diet composition between populations of the same species. While some species may specialize so heavily that the absence of favored prey items is enough to limit distributions (Pianka & Parker, 1975), other, more opportunistic feeders may have substantially different diets based on local prey availability between sites, even when those species are dietary generalists overall. Studies that incorporate diet analyses of multiple populations across different environmental settings can further our understanding of how much dietary plasticity exists within species, how the structure of predator communities is influenced by the structure of prey communities, and how spatial variation in prey availability can influence the co‐occurrence of predator species (Taverne et al., 2019).

Recent studies using molecular approaches have revealed previously hidden diversity in animal diets (Gil et al., 2020; Kartzinel & Pringle, 2015). Though taxonomic databases are still incomplete, their utility for characterizing dietary composition is proven in cases where morphological identification of diet items is difficult or impossible (Taberlet et al., 2012). Morphological studies of stomach contents can also be biased by the different rates of digestion between prey items based on size, hardness, and composition (Carretero, 2004). DNA metabarcoding for diet analysis using fecal matter is a technique that enables the identification of prey items without invasive methods, such as stomach flushing, bleeding, or specimen collection (Martínez‐Fonseca et al., 2022; Walker et al., 2016, 2019). For sensitive species or species of conservation concern, it remains the most promising avenue for understanding dietary diversity.

We used DNA metabarcoding to investigate the diets of three congeneric lizard species inhabiting the Madrean Sky Islands region in southeastern Arizona. The striped plateau lizard, *Sceloporus virgatus*, is a small‐to‐medium‐bodied habitat generalist that utilizes a variety of low perches, from small rocks and logs to dwelling on the ground (Smith, 1996). Slevin's bunchgrass lizard, *S. slevini*, is also small‐bodied, though more elongate, has reduced limbs compared to *S. virgatus*, and is almost exclusively grass‐dwelling (Ballinger & Congdon, 1981). These two species are narrowly allotopic in this system but overlap in spatial niche and ecomorphological space (Westeen et al., in press). Yarrow's spiny lizard, *S. jarrovii* is a medium‐to‐large lizard that is strongly saxicolous and occasionally arboreal (Simon & Middendorf, 1976). It is syntopic with the two smaller species but retains a distinct microhabitat and temporal niche from *S. slevini*; it overlaps somewhat spatially and temporally with *S. virgatus* (Westeen et al., in press). *Sceloporus virgatus* and *S. jarrovii* are sit‐and‐wait predators (Watters, 2009; Weiss, 2001); foraging habits for *S. slevini* have not been recorded but likely also conform to sit‐and‐wait predation given their shy nature and affinity for bunchgrass clusters (EPW, personal observation). Given the differences in spatiotemporal niche use among these species and their sedentary predation habits, we predict that interspecific dietary niche partitioning will be evident. More specifically, we predict that *S. slevini* will have the narrowest dietary niche due to its high habitat‐specificity and will overlap more in dietary niche space with *S. virgatus*, the other small‐bodied ground‐dweller, than it will with *S. jarrovii*. We collected fecal samples from 228 lizards from the Chiricahua Mountains and Appleton‐Whittell Research Ranch, Arizona, USA to examine how diet composition varies among these three species and between populations within each species. We then quantified intraspecific and interspecific niche breadth and compositional overlap to understand how these lizards utilize this important resource axis and how dietary niche partitioning may contribute to species interactions in syntopy.

## 2 METHODS

### 2.1 Field surveys

**FIGURE 1:**
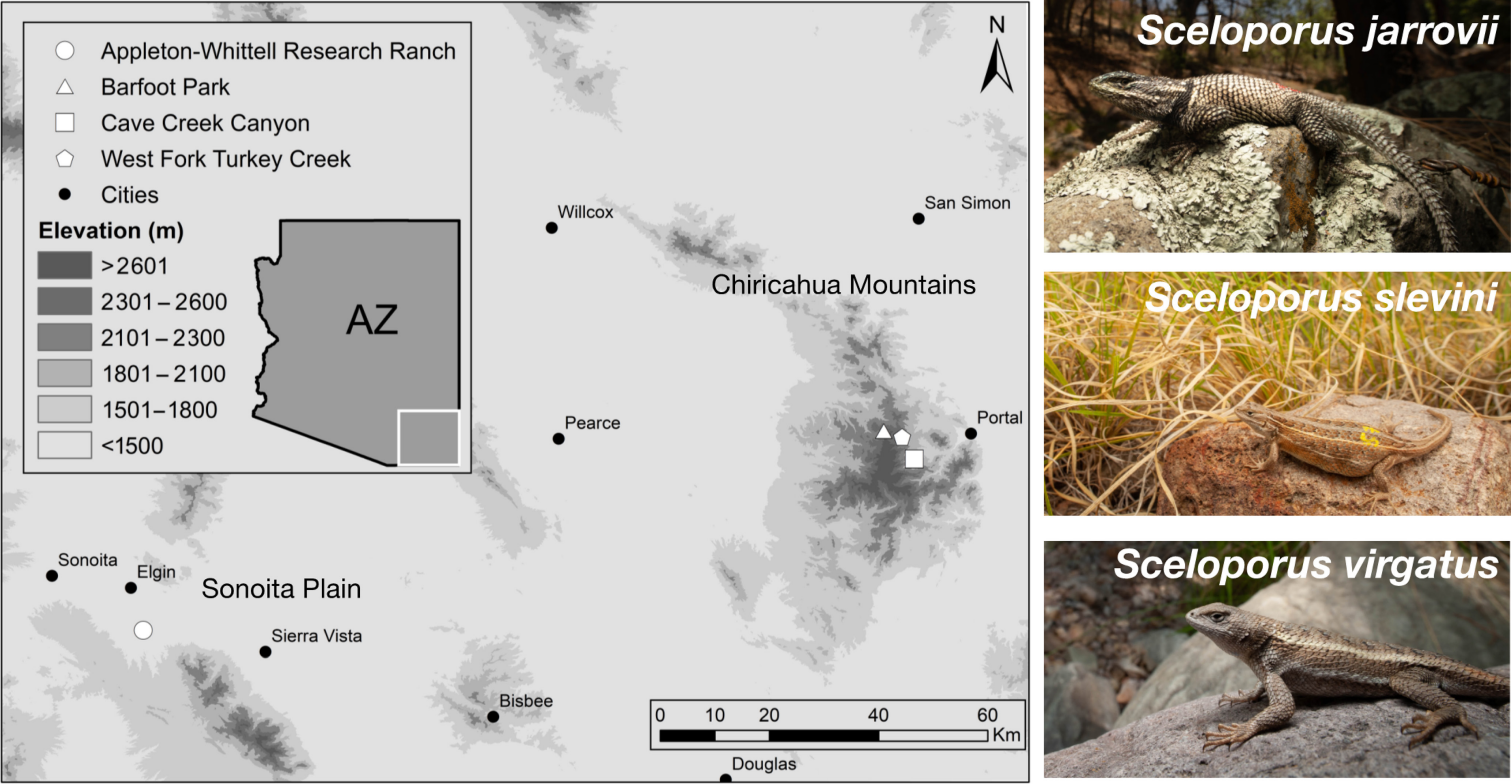
Study system including three sites in the Chiricahua Mountains and one in the Sonoita Plain, AZ, USA. Focal species are depicted to the right: *Sceloporus jarrovii* is a large‐bodied saxicolous species, *S. slevini* is a small‐bodied grass dweller, and *S. virgatus* is a small‐to‐medium terrestrial generalist.

We collected fecal samples from adult individuals of *Sceloporus jarrovii*, *S. slevini*, and *S. virgatus* in the Chiricahua Mountains and Appleton‐Whittell Research Ranch, AZ from 2019 to 2022 (Figure 1, Table 1). Sites within the Chiricahua Mountains included Cave Creek Canyon, comprised of Madrean Oak Woodland habitat; Turkey Creek, within the Madrean Pine‐oak habitat band; and Barfoot Park, an area of Montane Conifer Forest near the highest peaks of this mountain range. The Appleton‐Whittell Research Ranch (AWRR) in the Sonoita Plain, AZ, is a semi‐desert grassland that supports relict populations of *S. slevini* (Bock et al., 1990; d'Orgeix et al., 2011; Smith et al., 1998). Despite the relatively long geographic distance between these two sites, they represent two of the closest habitat patches for *S. slevini* in this region, as this species exhibits a very disjunct range overall (Watkins‐Colwell et al., 2003).

**TABLE 1:** Samples included in the study, by species and population.

Species	Site	Habitat	Elevation (m)	No. of samples
*S. jarrovii*	W. Fork Turkey Creek, Chiricahua Mtns, Cochise County, AZ	Madrean Pine‐oak	2070	42
*S. jarrovii*	Barfoot Park, Chiricahua Mtns, Cochise County, AZ	Montane Conifer Forest	2505	39
*S. slevini*	Barfoot Park, Chiricahua Mtns, Cochise County, AZ	Montane Conifer Forest	2505	38
*S. slevini*	Appleton‐Whittell Research Ranch, Sonoita Plain, Santa Cruz County, AZ	Semi‐desert Grassland	1430	32
*S. virgatus*	Cave Creek Canyon, Chiricahua Mtns, Cochise County, AZ	Madrean Oak Woodland	1700	44
*S. virgatus*	W. Fork Turkey Creek, Chiricahua Mtns, Cochise County, AZ	Madrean Pine‐oak	2070	33

*Note*: Each species is represented by two populations with paired low‐ and high‐elevation sites.

Lizards were captured by hand or lasso and processed in the field. Individuals were given a unique mark and released at their point of capture to ensure that they were not resampled for this study. Through intensive sampling efforts, we were able to exceed a target of 20 individuals per population (Rato et al., 2022) for all of our studied populations (Table 1). Lizards were captured across the active season (April–September) to document a summary of total spring–summer diet. Samples were collected directly from the cloaca and placed into RNALater or ethanol for preservation. Many lizards will defecate when captured, but in some cases, it was necessary to gently palpate lizards by hand or using a piece of foam following McGee et al. (2019) to induce defecation. Animal care and field surveys were approved by the University of California Berkeley and Virginia State University Institutional Animal Care and Use Committees (Protocol AUP‐2019‐02‐11,797 to EPW at UC Berkeley; Protocol 2017‐100 to CAD at Virginia State University), collection permits were issued by Arizona Game and Fish Department (LIC#SP653941, SP404320, SP407158, SP808336 to EPW; LIC#SP652734 to CAD), and land access was granted by Coronado National Forest, Douglas Ranger District.

### 2.2 Sample processing

We pooled fecal samples by population, resulting in six sample pools (Table 1). Pooled fecal samples were processed at Northern Arizona University's Pathogen and Microbiome Institute. We extracted genomic DNA using a QIAamp Fast DNA Stool Mini kit (Qiagen) following the human DNA analysis protocol, allowing lysis to occur for 30 min at 70°C, and then eluting DNA to 100 μL. To target arthropods, we amplified a short section (~185 bp insert) of cytochrome oxidase subunit I (COI) using the ANML primer set (forward: LCO1490, reverse: CO1‐CFMRa; Jusino et al., 2019). Primers were premodified with 5′ universal tails (Colman et al., 2015) for preparing sequencing libraries in a later PCR step. The first PCR was run in 15 μL reaction volumes with 3 μL of genomic DNA, 8.46 μL of PCR‐grade water, 1.5 μL 10× Mg‐free PCR buffer (Invitrogen, Thermo Fisher Scientific), 1.5 mM MgCl_2_, 0.2 mM each dNTP, 0.2 μM each primer, 0.16 μg/μL bovine serum albumin (Ambion Ultrapure BSA), and 0.03 U/μL PlatinumTaq DNA polymerase (Invitrogen, Thermo Fisher Scientific). We also included a negative template control (NTC) whereby PCR‐grade water was added as template to a reaction instead of genomic DNA. Thermal cycling included initial denaturation at 94°C for 5 min, 5 cycles of 94°C for 1 min, 45°C for 1.5 min, and 72°C for 1 min, 35 cycles of 94°C for 1 min, annealing at 50°C for 1.5 min, and 72°C for 1 min, with a final extension cycle of 72°C for 5 min. PCR product was subsequently used as template to a second PCR to add unique 8 bp indices for dual indexed, paired‐end sequencing and to make the amplicon flow‐cell ready (Colman et al., 2015). An index was only used once per sample. Reactions were run in 25 μL volumes with 2 μL amplicon template, 12.5 μL 2× Kapa HiFi HotStart ReadyMix (Roche Sequencing), 8.5 μL PCR‐grade water, and 1 μL each index primer (10 μM initial concentration). Thermal cycling conditions included an initial denaturation at 98°C for 2 min, followed by 8 cycles of 98°C for 30 s, 60°C for 20 s, and 72°C for 5 min, concluding with a final extension step of 72°C for 5 min. Amplified PCR product was then sequenced on an Illumina MiSeq V2 Micro 300 cycle kit with 30% PhiX with 3.5 pM of the pooled amplicon libraries.

Sequencing reads were processed in QIIME2 v2022.2 (Bolyen et al., 2019). Priming regions were removed using cutadapt v4.0 (Martin, 2011) to isolate the fragment of interest. Using DADA2 (Callahan et al., 2016), we removed low‐quality reads, denoised and merged paired‐end reads, and then filtered out PCR chimeric reads. DADA2 was run with both R1 and R2 reads truncated to 125 bp and with the expected error parameter (‐‐p‐max‐ee‐f, ‐‐p‐max‐ee‐r) set to 4.0. Amplicon sequence variants were then postclustered de novo into operational taxonomic units (OTUs) using Vsearch v2.7.0 (Rognes et al., 2016) at 98.5% similarity (O'Rourke et al., 2021). OTUs were cross‐referenced against the National Center for Biotechnology Information's (NCBI) GenBank database (Benson et al., 2009) using BLAST (Altschul et al., 1990), classified to phylum using least common ancestor (LCA) assignment in MEGAN v6 (Huson et al., 2007), and only OTUs assigned to Arthropoda and Chordata were retained for analysis (Sanchez, 2021). Although the focus of our study was on diet, the ANML primers may also co‐amplify host COI sequences and can allow for host verification in a fecal sample. Arthropod and chordate OTUs were then classified using a naïve‐Bayes machine learning classifier (Bokulich et al., 2018) that was trained against a previously validated reference library (O'Rourke et al., 2020, 2021). The reference library (“fullCOI_db” available at https://osf.io/qju3w/files/osfstorage) consists of all available invertebrate and vertebrate COI sequences assembled from the Barcode of Life Database (Ratnasingham & Hebert, 2007) and NCBI GenBank (Benson et al., 2009). The reference library was already trimmed to the ~185 bp ANML insert and made nonredundant through LCA (described here: https://github.com/devonorourke/tidybug/). We retained classifications above a threshold of 70% bootstrap support (O'Rourke et al., 2021). The complete OTU table may be found in Appendix S1.

### 2.3 Existing and novel diet records

We tabulated existing diet records for adult lizards of our three study species from the literature. We recorded results from any study that identified diet items for any of the three species (Ballinger & Ballinger, 1979; Barbault et al., 1985; Bergeron & Blouin‐Demers, 2020; Gadsden et al., 2011; Goldberg & Bursey, 1990; Simon, 1975; Watters, 2008). We also consulted field guides for the region (Degenhardt et al., 1996; Holycross et al., 2022; Jones & Lovich, 2009), which corroborated data from the literature but generally did not add records. Existing diet records may be found in Appendix S2. We did not consider studies in which lizards were fed or had their diets supplemented, nor did we consider diets of neonate lizards, which can differ significantly from adult conspecifics (Watters, 2010).

We cross‐referenced OTU identification with known arthropod records from the area during the spring and summer (May–August), which matches the sampling period of our study (Ballinger & Ballinger, 1979; Simon, 1975; Watters, 2010). Simon (1975) sampled both available arthropods and lizard prey items and found that all available prey types were ingested over the season with the exception of Neuropterans (net‐winged insects). We, therefore, used the total composition of prey items ingested by the three species as a proxy for available prey items in the environment. We identified all OTUs to the lowest taxonomy possible based on reference libraries. For comparisons of dietary breadth and composition, we used both the complete set of OTUs as well as a subset of OTUs that we were able to identify to order level. Evidence for whether sequence (read) numbers are interpretable as abundances is mixed but this process is generally discouraged as there are many potential factors affecting how much DNA results from prey items that are independent of prey biomass (Clare, 2014; Deagle et al., 2019; Di Muri et al., 2020; Lamb et al., 2019). Therefore, we evaluated diet items, based on OTUs, as either present or absent in each pooled diet sample based on whether they were found in the amplicon reads for the pool by our OTU identification workflow.

### 2.4 Inter and intraspecific niche breadth and overlap

We calculated total dietary niche breadth for each species by pooling the two populations we sampled per species and calculating Levin's index of niche breadth, $\mathrm{Bn}\left[j\right]=\frac{1}{\frac{R}{\sum \left(p{\left[i\right]}^{2}\right)}}$, where *R* is the number of different environments and p[*i*] is the proportion of taxon *j* in environment *i* (Levins, 1968). Following Pianka (1986), we consider the lizards as the ‘environments’ and the available food items as the taxa. The proportion of prey items was calculated as the number of prey OTUs present in each lizard species' diet compared to the total OTUs for all three species. We first used all prey OTUs to calculate breadth and overlap metrics; then we used only the subset of prey items we were able to identify to order level. To convert niche width to a standardized scale from 0 to 1 (specialist to generalist, respectively), we used the following equation: $B_{A}=\frac{\mathrm{Bn}\left[j\right]–1}{R–1}$. We also calculated niche width using the Shannon–Weiner Diversity Index: $H^{\prime }=-\mathrm{sum}\left(p_{j}\;\log\;p_{j}\right)$, where p_*j*_ is the proportion of samples containing resource *j* (Colwell & Futuyma, 1971). We then standardized the measure as $J^{\prime }=H^{\prime }/\log\left(n\right)$. We chose these two indices to provide complementary measures of niche breadth; Levin's index gives more weight to common resources used, while the Shannon–Weiner Index weights rare resources more heavily. For dietary niche breadth, the use of Levin's index of niche breadth is largely advocated over other indices (Hurlbert, 1978), so we base most of our discussions around this metric. We then compared diet breadth at OTU and order resolution between species using Kruskal–Wallis tests and Dunn tests for post hoc analyses, where appropriate (Van Den Berge et al., 2022).

We calculated niche overlap based on dietary composition between species using MacArthur and Levin's index $M_{\mathrm{jk}}=\frac{\mathrm{sum}\left(p_{\mathrm{ij}}\;p_{\mathrm{ik}}\right)}{\mathrm{sum}{\left(p_{\mathrm{ij}}\right)}^{2}}$, where *M*_*jk*_ is the overlap of species *k* on species *j*, p_*ij*_ is the proportion of resource *i* relative to the total resources used by species *j*, p_*ik*_ is the proportion of resource *i* out of the total resources used by species *k*, and *n* is the total number of resource states (MacArthur & Levins, 1967). We also calculated Pianka's index, *O*_*jk*_ = *O*_*kj*_ = $\frac{\mathrm{sum}\left(p_{\mathrm{ij}}*p_{\mathrm{jk}}\right)}{\surd \left(\mathrm{sum}\left({\left(p_{\mathrm{ij}}\right)}^{2}\right)\mathrm{sum}\left({\left(p_{\mathrm{jk}}\right)}^{2}\right)\right)}$ for total dietary overlap between species (Pianka, 1973), where p_*i*_, p_*j*_, and p_*k*_ are the same as in MacArthur and Levin's index. We compared dietary composition among species using a *Χ* ^2^ test with Monte Carlo simulation using 2000 replicates (Clare et al., 2014).

We then calculated niche breadth and overlap using these metrics for the interacting populations at two specific sites, Turkey Creek and Barfoot Park. Finally, we compared dietary composition between the two sites (populations) for each of the three species using Pianka's niche overlap metric. We assessed whether populations had different dietary compositions using *Χ* ^2^ tests with Monte Carlo simulation using 2000 replicates (Clare et al., 2014).

## 3 RESULTS

### 3.1 Sample processing

None of the negative controls were prepared with our samples amplified. We obtained 120,704 paired raw‐end reads (mean = 20,117.22, SD = 3091.01); after cleaning and retaining only arthropods and chordates, 105,996 reads remained (mean = 17,666, SD = 4799.12). We detected 53 unique OTUs across all levels of biological organization among our six sample pools (which each contained 32–44 individual lizard samples; Table 1), including some co‐amplification of the host species, which was excluded, for a total of 51 prey OTUs. 42 OTUs were identifiable to order level and spanned 8 orders including Araneae, Coleoptera, Diptera, Hemiptera, Hymenoptera, Isopoda, Lepidoptera, and Orthoptera. 32 OTUs were identified to family level, 21 were identified to genus level, and 10 were identified to species level (Appendix S1). Due to incomplete genetic reference libraries for this taxonomic group (arthropods), we cross‐checked the classifications against existing records of arthropod taxa and found that all identified OTUs represent taxa present in the study area. Furthermore, all identifiable OTUs matched existing prey records for these lizards at order level except for two; Watters (2008) documented termites (Order Blattodae, infraorder Isoptera) and Simon (1975) identified a gastropod, both in the stomach of *S. jarrovii* individuals, which were not present in our samples (Appendix S2). Only one family uncovered in this study has been identified previously: formicid ants were present in the diets of *S. jarrovii* and *S. virgatus* (Gadsden et al., 2011; Watters, 2008). Some records mentioned lower taxonomy by common name only (e.g., ‘spiders’; Appendix S2).

### 3.2 Interspecific niche breadth and overlap

**FIGURE 2:**
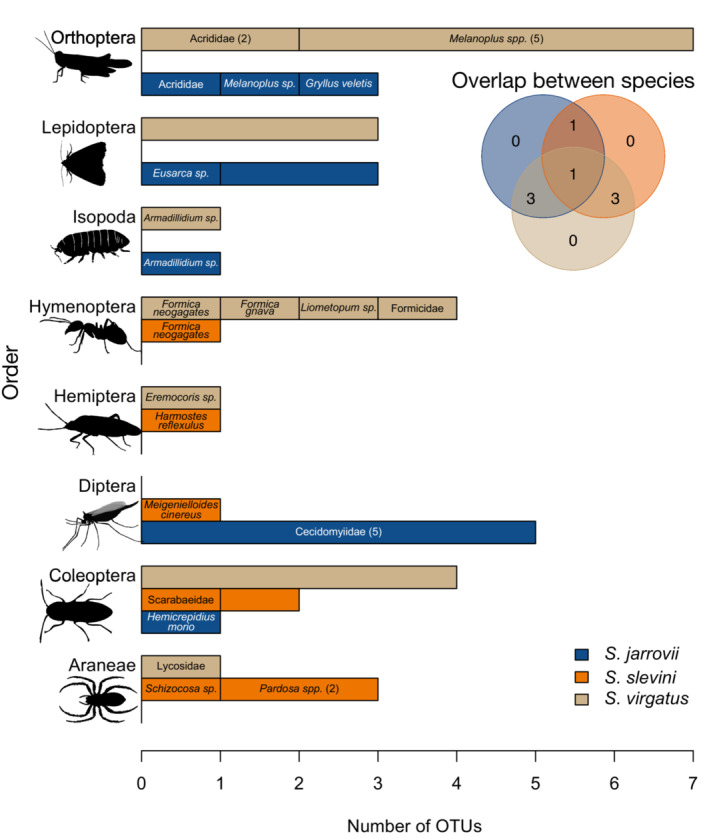
Diet items recovered in this study as given by the number of OTUs per prey order. The height of each colored segment represents the number of OTUs identified within the diet of each lizard species. When family, genus, or species‐level identification was possible from OTUs, those taxa are listed within the corresponding bar unit. Numbers in parentheses indicate the number of OTUs corresponding to that category. Bar units without text indicate OTUs that we were not able to identify past order level. *Sceloporus virgatus* consumed all prey orders but one and shows substantial overlap with the other two species, while *S. jarrovii* and *S. slevini* overlap in only two prey orders. Inset Venn diagram shows summarized overlap between the three species at order level.

Our study species differed significantly in dietary niche breadth by OTU (*Χ* ^2^ = 11.137, *p* = .003), with *S. virgatus* having the greatest niche breadth compared to *S. jarrovii* (*Z* = 2.66, *p* = .015) and *S. slevini* (*Z* = 3.073, *p* = .006; Table 2). *Sceloporus jarrovii* and *S. slevini* did not differ significantly in niche breadth (*Z* = 0.409, *p* = .682) despite having different dietary compositions (Table 2, Figure 2). When we analyzed only OTUS we could resolve to the order level, *S. virgatus* still had the greatest niche breadth (Table 2), but this was not statistically significant (*Χ* ^2^ = 2.574, *p* = .2761).

**TABLE 2:** Total dietary niche breadth for the three species (populations pooled) based on OTU identification and order and included in the study.

Species	Std. Levin's index (OTU)	Std. Shannon's index (OTU)	Std. Levin's index (order)	Std. Shannon's index (order)
*S. jarrovii*	0.28	0.688	0.393	0.692
*S. slevini*	0.24	0.652	0.429	0.718
*S. virgatus*	0.54	0.847	0.534	0.822

*Note*: *Sceloporus virgatus* exhibits the most dietary generalism, as indicated by the largest niche width across all metrics.

**FIGURE 3:**
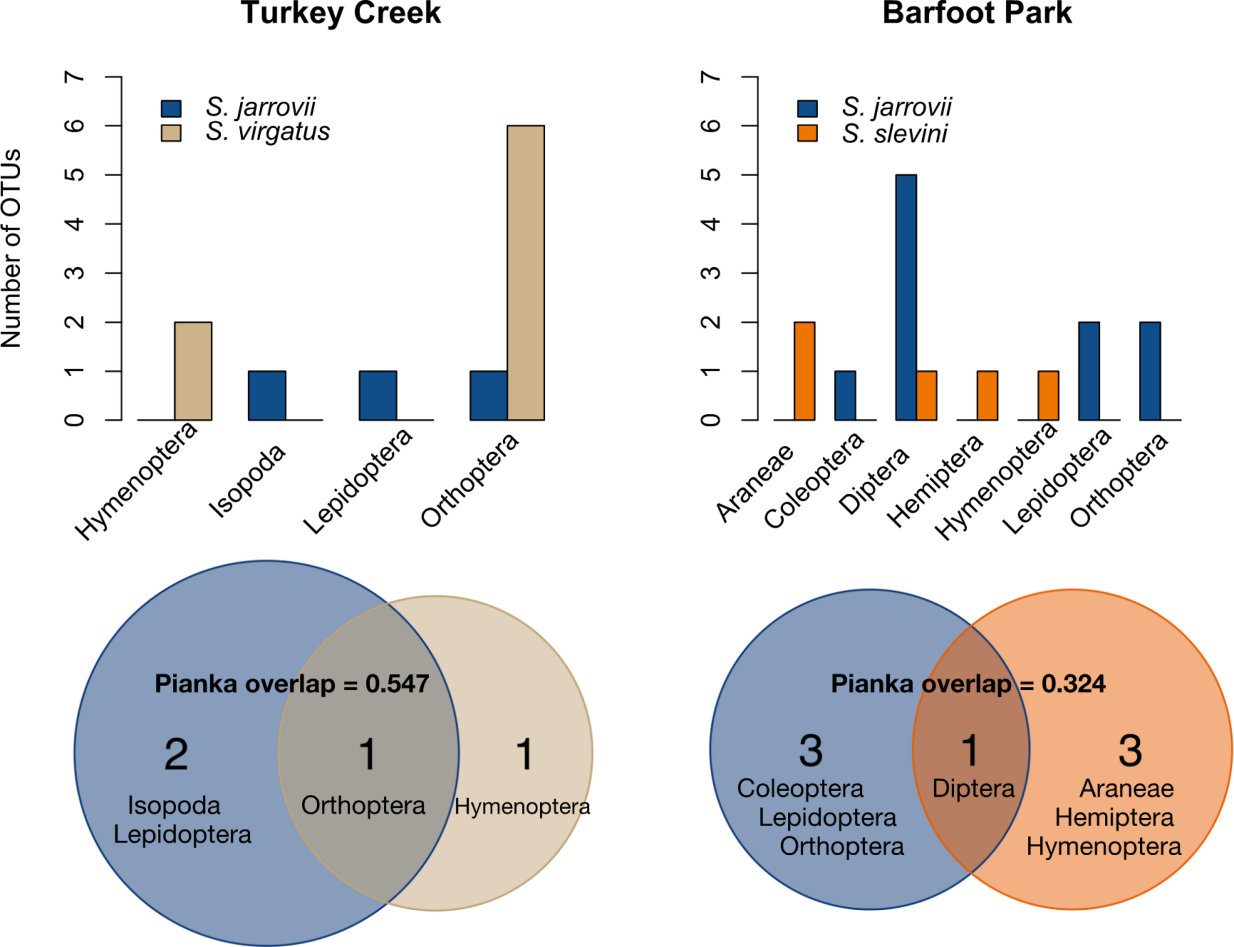
Site‐specific comparisons reveal dietary niche partitioning between the two sets of syntopic species. Top: Bar height represents number of OTUs identified from each species corresponding to that order. Bottom: Venn diagrams show the number of diet categories by order unique and shared between sets of interacting species.

Species differed significantly in dietary composition at the order level (*Χ* ^2^ = 29.926, *p* = .0134). Compositional niche overlap was highest between the two more generalist species, *S. virgatus* and *S. jarrovii* (Figure 3, Table 3), and lowest between *S. jarrovii* and *S. slevini*, both in terms of overall dietary composition (Table 3) and site‐specific diets where they co‐occur at Barfoot Park (Figure 3). In this system, *Sceloporus slevini* and *S. virgatus* are narrowly allotopic; despite not occurring at the same sites, they had moderate dietary overlap (Table 3).

**TABLE 3:** Dietary niche overlap by OTU and prey order for each pair of species included in the study.

Species pairs	Pianka's niche overlap (OTU)	Levin's niche overlap (OTU)	Pianka's niche overlap (order)	Levin's niche overlap (order)
*S. jarrovii–S. virgatus S*yntopic	0.195	0.143	0.541	0.481
*S. jarrovii–S. slevini* Syntopic	0.000	0.000	0.261	0.252
*S. slevini–S. virgatus* Allotopic	0.052	0.035	0.415	0.381

*Note*: *Sceloporus virgatus* overlaps more with *S. jarrovii* and with *S. slevini* than *S. jarrovii* and *S. slevini* do with one another.

### 3.3 Intraspecific niche overlap

**FIGURE 4:**
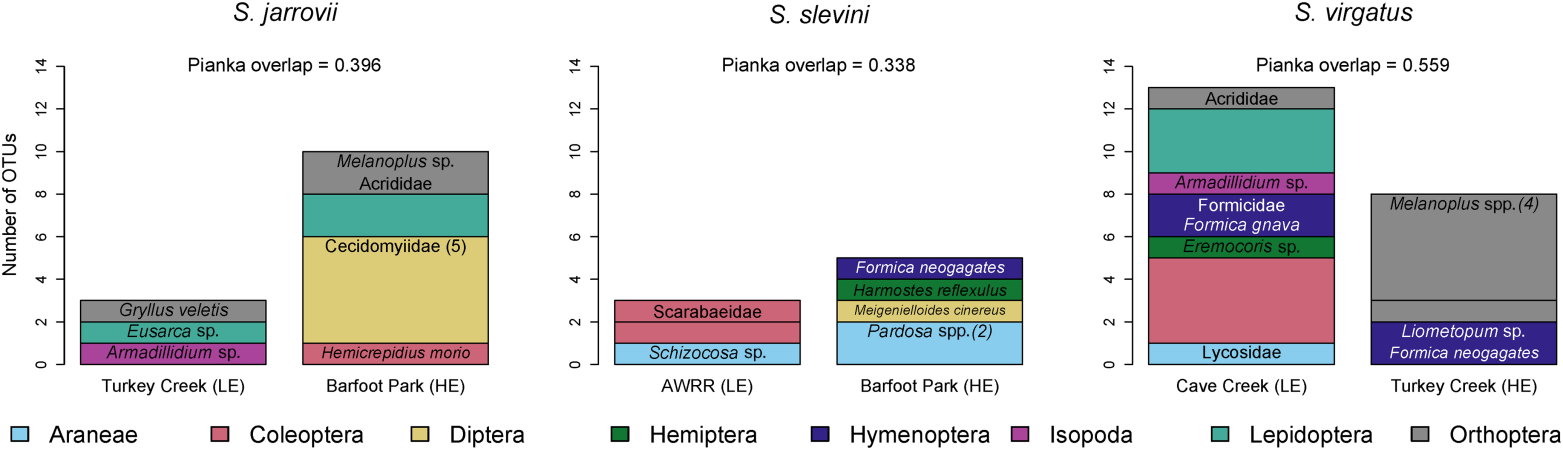
Colored bar height corresponds to the number of OTUs per order within the diet of each population. Unlabeled bars indicate OTUs unable to be identified beyond order level. Lizard diets vary by population; all species exhibit differences between populations, but the greatest differences are observed for *S. slevini*, followed by *S. jarrovii* and then *S. virgatus*. For each species, its respective low‐elevation site (LE) is plotted to the left and high‐elevation site (HE) to the right. AWRR, Appleton–Whittell Research Ranch.

Populations within species differed in dietary composition, though not significantly (*Χ* ^2^ = 9.4735, *p* = .096). *Sceloporus slevini* had the least dietary overlap between its two sites, followed by *S. jarrovii*; *S. virgatus* had the highest level of overlap (Table 4). For *S. jarrovii* and *S. slevini*, high‐elevation populations (Barfoot Park, 2505 m) revealed greater dietary richness compared to low‐elevation sites despite similar sample sizes (Figure 4).

**TABLE 4:** Dietary niche overlap by prey order between the two populations for each species.

Species	Pianka's niche overlap (order)	Levin's niche overlap (order)
*S. jarrovii*	0.396	0.4
*S. slevini*	0.338	0.24
*S. virgatus*	0.559	0.492

*Note*: Populations did not share any OTUs between sites but shared multiple diet items at order level.

### 3.4 Novel diet records

Our results introduce more specificity into the identification of prey categories: previously, the vast majority of records were identified only to order level (Appendix S2). The following families that we detected have not been identified previously by name in the diets of these lizards: for *S. jarrovii* Acrididae, Armadillidiidae, Cecidomyiidae, Elateridae, Geometridae, and Gryllidae; for *S. slevini* Formicidae, Lycosidae, Rhopalidae, Scarabaeidae, and Tachinidae; and for *S. virgatus* are Acrididae, Armadillidiidae, Lycosidae, and Rhyparochromidae. Additionally, all of the records we identified to the genus or species levels are novel for these lizard species. Novel records are indicated in Appendix S1.

## 4 DISCUSSION

With the use of DNA metabarcoding, we recovered a great deal of dietary richness, including previously unreported families, genera, and species, in the diets of three *Sceloporus* lizard species in southeastern Arizona. By using samples taken across the spring and summer, we obtained a dietary summary during a period of prey abundance. We found evidence for dietary niche partitioning between interacting species as well as intraspecific differences in diet between populations.

### 4.1 Interspecific niche breadth and overlap

Dietary breadth and composition varied between the three species (Table 2, Figure 2), providing evidence that dietary partitioning may structure interactions in this system. Previous work on the diets of *S. virgatus* and *Urosaurus ornatus*, two lizards similar in size and ecology, found very few differences in diet (Bergeron & Blouin‐Demers, 2020). The differences in diet we uncovered between the three *Sceloporus* species match what is known about niche partitioning more generally in this system. We previously showed that perch height and type were significantly different between species in this system (Westeen et al., in press). Given the nature of these species as sit‐and‐wait predators, it follows that these microhabitat differences lead to different availability of prey, which in turn contribute to the dietary differences we observed. Evidence for optimal foraging theory is limited in lizards, including explicit studies of these species (Stamps et al., 1981; Watters, 2010); lizards generally eat prey items in relation to their availability in this system. Therefore, we suspect that most differences in diet in this system are due to differences in microhabitat and localized prey availability. Contrary to our prediction that the two small, ground‐dwellers—*S. slevini* and *S. virgatus—*would overlap most in diet composition, we found the highest overlap between *S. jarrovii* and *S. virgatus* (Table 3). These two species are more generalist in their habitat as they occupy perches from the ground level up into trees and rocks, perhaps providing more opportunities for the two species to overlap in foraging areas. However, the overlap between *S. slevini* and *S. virgatus* was similar; these two species have very similar spatial niches (Westeen et al., in press), and their dietary niche overlap is consistent with this. This similarity may limit their ability to co‐exist, and as such they are narrowly allotopic in this system. We also found that *S. virgatus* had the broadest dietary niche width of our three study species (Table 2), consistent with a role as a generalist predator. Furthermore, we found that the dietary niche of *S. virgatus* overlapped with the two other species more than they did with one another (Figure 4). This supports the idea that *S. slevini* and *S. jarrovii* maintain distinct dietary niches from one another, while *S. virgatus* exhibits a broad dietary niche that encompasses some of the dietary diversity of both *S. jarrovii* and *S. virgatus*.

Analyses at OTU and order levels provide similar but complementary information. For instance, niche breadth at OTU resolution suggests that *S. jarrovii* feeds more broadly than *S. slevini*, whereas at order level, we observed the opposite pattern (Table 2). Different OTUs may represent the same taxa and, therefore, overestimate measures of richness and breadth while underestimating dietary overlap. Yet, only using prey items to the order level can sacrifice specificity and, thus, underestimate the degree of dietary partitioning occurring in this system. For example, prey items in the same order can vary substantially in size and ecology, such as small‐bodied weevils and large Scarab beetles that are both Coleopterans, further contributing to dietary preferences and partitioning. Previous work in this system has shown that gape width is related to prey‐size selection (Bursey & Goldberg, 1993); though prey size is not an aspect of the current study, future work may consider the relationship between individual‐level diet and predator ecomorphology and how size selection of prey may reduce interspecific competition as it does intraspecific competition (Simon, 1976). As taxonomic databases continue to grow, analyses at OTU resolution will provide the most complete dietary information; until then, subsetting OTUs to those which can be identified to a more ecologically pertinent group, such as family or order, remains a useful addition to OTU‐level analyses.

### 4.2 Intraspecific niche overlap

We also uncovered differences in diet composition between populations of the same species (Figure 4), though they were not statistically significant. *Sceloporus slevini* is a microhabitat specialist and exhibited the least dietary overlap between sites (Pianka overlap = 0.338): the two sites are geographically distant (121 km straight‐line distance), situated at different elevations (Table 1), possess markedly different vegetation (semi‐desert grassland vs. montane conifer forest), and have different temperature regimes. Given these differences in habitat, populations may have very different access to prey communities between sites. Despite also being the smallest species and the species with the narrowest gap width per body size (Westeen et al., in press), spiders and especially wolf spiders in the family Lycosidae appeared in the diet *S. slevini* at both sites (Figure 4). Existing studies on the diet of *S. slevini* are rare; Newlin (1974) found hemipterans and ants to be the most significant diet categories by volume. Barbault et al. (1985) found beetles, ants, hemipterans, and grasshoppers to contribute significantly to diets in Durango, Mexico, though given current taxonomy and distributions, it is possible that these results do not represent *S. slevini* but another member of the *S. scalaris* group, *S. brownorum* (Grummer & Bryson, 2014). Neither report spiders as contributing significantly to the diet of this species; observational studies would be a welcome follow‐up to understand how often spiders are consumed.

For *S. jarrovii* the two sites we sampled are in close geographic proximity (3.3 km straight‐line distance), yet population‐level diet overlap (Pianka overlap = 0.396) is similar to that of *S. slevini* (Pianka overlap = 0.338), which had substantially more distance between populations. We previously uncovered differences in microhabitat use by *S. jarrovii* between these sites (Westeen et al., in press), which may contribute to the dietary divergence between populations. Previous works report Hymenopterans, especially ants, as major diet items (Barbault et al., 1985; Goldberg & Bursey, 1990; Watters, 2008). Formicid ants were present at both sites occupied by *S. jarrovii* but not consumed; they were consumed by *S. slevini* and *S. virgatus*, however (Figure 4), perhaps serving as evidence of a competitive effect or a difference in prey availability in each species' preferred microhabitat.

Taken together, our findings on the dietary niche breadth and overlap between populations in *S. jarrovii* and *S. slevini* suggest that although they exhibit very similar levels of dietary niche breadth at the species level (Table 2) and population‐level diet overlap within each species (Table 4), their diet composition is structured in very different ways (Figures 2 and 3). Analyses of dietary niche that are conducted only at the species level may overlook important differences in how diet composition varies between populations.

The most habitat‐ and dietary‐generalist, *S. virgatus,* reveals greater dietary overlap between sites than the other two species (Table 4, Figure 4). With the greatest overall dietary niche width, it may be easier to find overlap between populations given the sheer number of diet items consumed at each site. However, we do see two categories that stand out as relatively important in the diet for this species at both sites: Hymenopterans, namely ants, and Orthopterans, namely grasshoppers. Previous work underscores the importance of Hymenopterans as a prey item; Bergeron and Blouin‐Demers (2020) found that they comprise >75% of prey items consumed, while Watters (2008) found that formicid ants comprised about 50% of observational consumptions and 30% of stomach contents.

### 4.3 Novel diet records

The dietary diversity uncovered in this study complements previous work that examined prey items from the stomachs of the three species herein (Appendix S1). The use of metabarcoding allowed us to achieve finer resolution of prey identification in most instances, while avoiding stomach flushing that can potentially impact the health of lizards, especially of the small‐bodied *S. slevini* that has already suffered severe population reductions at both sites herein (Ballinger & Congdon, 1996; Bock et al., 1990; d'Orgeix et al., 2011; Smith et al., 1998). An interesting next step would be to pair observational studies or microscopic identification with metabarcoding to further understand how size selection of prey—an important factor at least for *S. jarrovii* (Simon, 1976) and likely for the other species as well—structures diets within and between species. We hope that the utility of DNA metabarcoding in this study inspires other researchers to employ this method to document prey items of lizards in different contexts.

## 5 CONCLUSIONS

The use of DNA barcoding enabled us to capture dietary breadth and composition of three lizards, including one species, *S. slevini*, for which other methods such as stomach flushing would be inadvisable due to their small size and sensitive nature. We document previously unknown diet items and reveal both interspecific and intraspecific dietary differences. Interspecific prey consumption appears related to differences in microhabitat and may contribute to patterns of sympatry between species. Future studies will benefit from comparisons between sexes, across seasons, from volumetric analyses of prey items to reveal relative abundance, and from prey‐size analyses to further illuminate the drivers of dietary niche partitioning in this system and among squamate species in general. Further, an understanding of interspecific dietary partitioning can provide critical information for resource managers to optimize the long‐term survival of these three species and serve as a template for other sympatric species.

## AUTHOR CONTRIBUTIONS

**Erin P. Westeen:** Conceptualization (lead); data curation (equal); formal analysis (equal); funding acquisition (lead); methodology (equal); project administration (equal); resources (equal); software (equal); visualization (lead); writing – original draft (lead). **José G. Martínez‐Fonseca:** Conceptualization (supporting); resources (equal); writing – review and editing (equal). **Christian A. d'Orgeix:** Resources (equal); writing – review and editing (equal). **Faith M. Walker:** Data curation (equal); formal analysis (equal); methodology (equal); resources (equal); software (equal); validation (equal); writing – review and editing (equal). **Daniel E. Sanchez:** Data curation (equal); formal analysis (equal); methodology (equal); resources (equal); software (equal); validation (equal); writing – review and editing (equal). **Ian J. Wang:** Conceptualization (supporting); funding acquisition (supporting); supervision (lead); visualization (supporting); writing – review and editing (lead).

## CONFLICT OF INTEREST STATEMENT

The authors declare no competing interests.

## Supporting information


Appendix S1:

## Data Availability

The original sequence data are available in the NCBI Sequence Read Archive under BioProject ID: PRJNA974030.
