# Supplementary material for: Dietary niche partitioning of three Sky Island Sceloporus lizards as revealed through DNA metabarcoding
Source: Ecol Evol. 2023 Sep 7;13(9):e10461. doi: 10.1002/ece3.10461 (PMC10485322; doi:10.1002/ece3.10461)
Supplement: Supplementary file 1 — Appendix S1: [file ECE3-13-e10461-s001.docx]

| **OTU** | **species** | **site** | **Reads** | **Kingdom** | **Phylum** | **Class** | **Order** | **Family** | **Genus** | **Species** | **Confidence** | **Novel Record?** |
| --- | --- | --- | --- | --- | --- | --- | --- | --- | --- | --- | --- | --- |
| 0bd57edc4a4ad22ac27632357e4def17 | jarrovii | Barfoot Park | 699 | Animalia | Arthropoda | Insecta |  |  |  |  | 0.99386538 |  |
| 295005dc12e26e8f6aefed7b48198cde | jarrovii | Barfoot Park | 502 | Animalia | Arthropoda | Insecta | Diptera | Cecidomyiidae | |  | 0.7489862 | Yes |
| 4eb0b234986a440903fd6a4c3a9f032f | jarrovii | Barfoot Park | 5676 | Animalia | Arthropoda | Insecta | Diptera | Cecidomyiidae | |  | 0.71904732 | Yes |
| 6b29a4dce3f636b9c05e67da14cdd7eb | jarrovii | Barfoot Park | 4992 | Animalia | Arthropoda | Insecta | Orthoptera | Acrididae |  |  | 0.99710404 | Yes |
| 70c4c90ea749392b59dc5212c56438fe | jarrovii | Barfoot Park | 802 | Animalia | Arthropoda | Insecta | Lepidoptera |  |  |  | 0.99896945 |  |
| a70cfa12868ac6293b10df2f755380f8 | jarrovii | Barfoot Park | 696 | Animalia | Arthropoda | Insecta | Diptera | Cecidomyiidae | |  | 0.99399696 | Yes |
| ad44856216676a34a6853367d40612fa | jarrovii | Barfoot Park | 131 | Animalia | Arthropoda | Insecta | Orthoptera | Acrididae | Melanoplus |  | 0.72435956 | Yes |
| b8598e1332ae55afdcebf495ed35b00e | jarrovii | Barfoot Park | 391 | Animalia | Arthropoda | Insecta | Diptera | Cecidomyiidae | |  | 0.83257579 | Yes |
| bbfb93b59d371997ccb94f3af996bf1a | jarrovii | Barfoot Park | 1115 | Animalia | Arthropoda | Insecta | Coleoptera | Elateridae | Hemicrepidius | Hemicrepidius morio | 0.9201617 | Yes |
| ec7411651bdf29559404df859e663fba | jarrovii | Barfoot Park | 321 | Animalia | Arthropoda | Insecta | Lepidoptera |  |  |  | 0.99905788 |  |
| f1efc0e5494db5cca3d581fe0bdf6e3a | jarrovii | Barfoot Park | 288 | Animalia | Arthropoda | Insecta | Diptera | Cecidomyiidae | |  | 0.98623537 | Yes |
| 24a346618d0d3d51734bbf958a561744 | jarrovii | Turkey Creek | 1048 | Animalia | Arthropoda | Insecta | Orthoptera | Gryllidae | Gryllus | Gryllus veletis | 0.99993817 | Yes |
| 7194586954a914b7328b10e07451d516 | jarrovii | Turkey Creek | 1780 | Animalia | Arthropoda | Insecta | Lepidoptera | Geometridae | Eusarca |  | 0.81088576 | Yes |
| d9f26f7fe23eecdf9750784201d78f73 | jarrovii | Turkey Creek | 676 | Animalia | Arthropoda | Insecta |  |  |  |  | 0.98465316 |  |
| eb19e14cd8f6abe2b9aeeb30216ae89f | jarrovii | Turkey Creek | 3554 | Animalia | Arthropoda | Malacostraca | Isopoda | Armadillidiidae | Armadillidium | Ambiguous_taxa | 0.83435202 | Yes |
| 263b9245da5aef3140bc9762059894d9 | slevini | Barfoot Park | 2 | Animalia | Arthropoda | Insecta | Diptera | Tachinidae | Meigenielloides | Meigenielloides cinereus | 0.99734723 | Yes |
| 5ec568e5376a96e9df3ccd697cbc9217 | slevini | Barfoot Park | 16363 | Animalia | Arthropoda | Arachnida | Araneae | Lycosidae | Pardosa |  | 0.97732963 | Yes |
| 66a898a5f51af39f4ff06a365fbe89db | slevini | Barfoot Park | 8 | Animalia | Arthropoda | Insecta | Hymenoptera | Formicidae | Formica | Formica neogagates | 0.85742937 | Yes |
| 8e0aebeacebf97794797c6043fd79dd5 | slevini | Barfoot Park | 56 | Animalia | Arthropoda | Arachnida | Araneae | Lycosidae | Pardosa |  | 0.97541415 |  |
| 99d21bb97db212cf1c5ced8f85e55774 | slevini | Barfoot Park | 95 | Animalia | Arthropoda | Insecta | Hemiptera | Rhopalidae | Harmostes | Harmostes reflexulus | 0.99999404 | Yes |
| 2e01ff7fef7f0e7c9c7602d6ac3920dd | slevini | AWRR | 130 | Animalia | Arthropoda | Insecta |  |  |  |  | 0.99010699 |  |
| 35e02f112758ec220f7b9e81a5e4fc1f | slevini | AWRR | 18731 | Animalia | Arthropoda | Insecta | Coleoptera |  |  |  | 0.87504736 |  |
| 4176432e46f86ede4f3dfce558d4f9f1 | slevini | AWRR | 92 | Animalia | Arthropoda | Insecta | Coleoptera | Scarabaeidae | |  | 0.82459249 | Yes |
| f90879c926c7a242604375eafa41f17c | slevini | AWRR | 8 | Animalia | Arthropoda | Insecta |  |  |  |  | 0.97935178 |  |
| fa521b61424e0e39230125eda2588c9f | slevini | AWRR | 2016 | Animalia | Arthropoda | Arachnida | Araneae | Lycosidae | Schizocosa | Schizocosa sp. 4GAB | 0.99651998 | Yes |
| 01f2d3ca1a6aec7a00a84404599ee44c | virgatus | Cave Creek | 43 | Animalia | Arthropoda | Insecta | Hymenoptera | Formicidae | Formica | Formica gnava | 0.99999241 | Yes |
| 0531bde514327cd638ed5e6855d78a73 | virgatus | Cave Creek | 86 | Animalia | Arthropoda | Insecta |  |  |  |  | 0.96887143 |  |
| 0c77fc6264a694c5e825558c0d3b4801 | virgatus | Cave Creek | 74 | Animalia | Arthropoda | Insecta | Coleoptera |  |  |  | 0.8200861 |  |
| 278d9973ea7d978eb81fa8ad0ac78300 | virgatus | Cave Creek | 109 | Animalia | Arthropoda | Insecta |  |  |  |  | 0.99023691 |  |
| 438fb4ed1877b9fc25b804b62c1844c1 | virgatus | Cave Creek | 54 | Animalia | Arthropoda | Insecta | Coleoptera |  |  |  | 0.86844236 |  |
| 6b29a4dce3f636b9c05e67da14cdd7eb | virgatus | Cave Creek | 23 | Animalia | Arthropoda | Insecta | Orthoptera | Acrididae |  |  | 0.99710404 | Yes |
| 70c4c90ea749392b59dc5212c56438fe | virgatus | Cave Creek | 511 | Animalia | Arthropoda | Insecta | Lepidoptera |  |  |  | 0.99896945 |  |
| 86325013b012cf7d19f096b9d45fecf3 | virgatus | Cave Creek | 47 | Animalia | Arthropoda | Insecta | Coleoptera |  |  |  | 0.86452186 |  |
| aad1e4f62d463dd1005df5ac4c68c59c | virgatus | Cave Creek | 3 | Animalia | Arthropoda | Insecta | Hymenoptera | Formicidae |  |  | 0.92241464 |  |
| bf35f8b93a7acb984e1f3ec70fb90150 | virgatus | Cave Creek | 56 | Animalia | Arthropoda | Insecta | Coleoptera |  |  |  | 0.77455135 |  |
| d31a4bc5742095355a51c95fc8419025 | virgatus | Cave Creek | 28 | Animalia | Arthropoda | Insecta |  |  |  |  | 0.97694626 |  |
| d8f31b46b68ef9808d2866192ebeee0b | virgatus | Cave Creek | 11 | Animalia | Arthropoda | Insecta | Lepidoptera |  |  |  | 0.99997293 |  |
| da6a2b1891e37f7bbc5f64558d4710d0 | virgatus | Cave Creek | 35 | Animalia | Arthropoda | Insecta | Lepidoptera |  |  |  | 0.99699357 |  |
| dd04ec8300c45d9c430ebda626467cc4 | virgatus | Cave Creek | 5 | Animalia | Arthropoda | Arachnida | Araneae | Lycosidae |  |  | 0.9994708 | Yes |
| de17f877a0189bd16268fbd63c2e9232 | virgatus | Cave Creek | 4 | Animalia | Arthropoda | Insecta | Hemiptera | Rhyparochromidae | Eremocoris |  | 0.99998078 | Yes |
| eb19e14cd8f6abe2b9aeeb30216ae89f | virgatus | Cave Creek | 72 | Animalia | Arthropoda | Malacostraca | Isopoda | Armadillidiidae | Armadillidium | Ambiguous_taxa | 0.83435202 | Yes |
| f6351cc4bde6dc9d036fb5a877df9e5d | virgatus | Cave Creek | 56 | Animalia | Arthropoda | Insecta |  |  |  |  | 0.9751321 |  |
| fb96dd37fee7479aad89a4ebef844142 | virgatus | Cave Creek | 23 | Animalia | Arthropoda | Insecta |  |  |  |  | 0.99921377 |  |
| 111a19cbe1f6279f8316043105712352 | virgatus | Turkey Creek | 412 | Animalia | Arthropoda | Insecta | Orthoptera | Acrididae | Melanoplus |  | 0.87360778 | Yes |
| 18ebc10927d57cbf41938c6fb7804118 | virgatus | Turkey Creek | 49 | Animalia | Arthropoda | Insecta |  |  |  |  | 0.96507811 |  |
| 1b4c27d238ebf447f17e1d82e705e88a | virgatus | Turkey Creek | 378 | Animalia | Arthropoda | Insecta | Orthoptera | Acrididae |  |  | 0.8508449 | Yes |
| 29176cb3573c4b670702044beb65b6d2 | virgatus | Turkey Creek | 127 | Animalia | Arthropoda | Insecta | Orthoptera | Acrididae | Melanoplus |  | 0.92687466 | Yes |
| 66a898a5f51af39f4ff06a365fbe89db | virgatus | Turkey Creek | 145 | Animalia | Arthropoda | Insecta | Hymenoptera | Formicidae | Formica | Formica neogagates | 0.85742937 | Yes |
| 6a17b6566c98277a6aac7c6e1bd28c9f | virgatus | Turkey Creek | 1777 | Animalia | Arthropoda | Insecta | Hymenoptera | Formicidae | Liometopum | | 0.9990825 | Yes |
| 9a098fffc34b01a84897211e4c54dcc6 | virgatus | Turkey Creek | 3826 | Animalia | Arthropoda | Insecta | Orthoptera | Acrididae | Melanoplus |  | 0.9818197 | Yes |
| ad44856216676a34a6853367d40612fa | virgatus | Turkey Creek | 2197 | Animalia | Arthropoda | Insecta | Orthoptera | Acrididae | Melanoplus |  | 0.72435956 | Yes |
| b9a72b44989c714390ee947262e54f59 | virgatus | Turkey Creek | 412 | Animalia | Arthropoda | Insecta | Orthoptera | Acrididae | Melanoplus |  | 0.95200837 | Yes |

Appendix 1: Diet records, as given by identified OTUs, recovered from fecal samples in this study.

| **species** | **life stage** | **Phylum** | **Subphylum** | **Class** | **Order** | **Family/Suborder** | **study location** | **method of ID** | **citation** |  |
| --- | --- | --- | --- | --- | --- | --- | --- | --- | --- | --- |
| Sceloporus virgatus | adult | Arthropoda | Hexapoda | Insecta | Coleoptera |  | Chiricahua Mtns, AZ -Middle Fork, Cave Creek | Manual ID of fecal pellets | Bergeron & Blouin-Demers 2020, Copeia |  |
| Sceloporus virgatus | adult | Arthropoda | Hexapoda | Insecta | Diptera |  | Chiricahua Mtns, AZ -Middle Fork, Cave Creek | Manual ID of fecal pellets | Bergeron & Blouin-Demers 2020, Copeia |  |
| Sceloporus virgatus | adult | Arthropoda | Hexapoda | Insecta | Hemiptera |  | Chiricahua Mtns, AZ -Middle Fork, Cave Creek | Manual ID of fecal pellets | Bergeron & Blouin-Demers 2020, Copeia |  |
| Sceloporus virgatus | adult | Arthropoda | Hexapoda | Insecta | Homoptera |  | Chiricahua Mtns, AZ -Middle Fork, Cave Creek | Manual ID of fecal pellets | Bergeron & Blouin-Demers 2020, Copeia |  |
| Sceloporus virgatus | adult | Arthropoda | Hexapoda | Insecta | Hymenoptera | | Chiricahua Mtns, AZ -Middle Fork, Cave Creek | Manual ID of fecal pellets | Bergeron & Blouin-Demers 2020, Copeia |  |
| Sceloporus virgatus | adult | Arthropoda | Hexapoda | Insecta | Lepidoptera | | Chiricahua Mtns, AZ -Middle Fork, Cave Creek | Manual ID of fecal pellets | Bergeron & Blouin-Demers 2020, Copeia |  |
| Sceloporus virgatus | adult | Arthropoda | Hexapoda | Insecta | Orthoptera |  | Chiricahua Mtns, AZ -Middle Fork, Cave Creek | Manual ID of fecal pellets | Bergeron & Blouin-Demers 2020, Copeia |  |
| Sceloporus virgatus | adult | Arthropoda | Chelicerata | Arachnida | Araneae |  | Chiricahua Mtns, AZ -Middle Fork, Cave Creek | Manual ID of fecal pellets | Bergeron & Blouin-Demers 2020, Copeia |  |
| Sceloporus virgatus | adult | Arthropoda | Crustacea | Malacostraca | Isopoda |  | Chiricahua Mtns, AZ -Middle Fork, Cave Creek | Manual ID of fecal pellets | Bergeron & Blouin-Demers 2020, Copeia |  |
| Sceloporus virgatus | adult | Arthropoda | Chelicerata | Arachnida |  |  | Chiricahua Mtns, AZ - John Hands and Herb Martyr CGs | Manual ID of stomach contents | Watters 2008, Herp. Review |  |
| Sceloporus virgatus | adult | Arthropoda | Hexapoda | Insecta | Coleoptera |  | Chiricahua Mtns, AZ - John Hands and Herb Martyr CGs | Manual ID of stomach contents | Watters 2008, Herp. Review |  |
| Sceloporus virgatus | adult | Arthropoda | Hexapoda | Insecta |  | Formicidae | Chiricahua Mtns, AZ - John Hands and Herb Martyr CGs | Manual ID of stomach contents | Watters 2008, Herp. Review |  |
| Sceloporus virgatus | adult | Arthropoda | Hexapoda | Insecta | Heteroptera | | Chiricahua Mtns, AZ - John Hands and Herb Martyr CGs | Manual ID of stomach contents | Watters 2008, Herp. Review |  |
| Sceloporus virgatus | adult | Arthropoda | Hexapoda | Insecta | Hymenoptera | | Chiricahua Mtns, AZ - John Hands and Herb Martyr CGs | Manual ID of stomach contents | Watters 2008, Herp. Review |  |
| Sceloporus virgatus | adult | Arthropoda | Hexapoda | Insecta | Lepidoptera | | Chiricahua Mtns, AZ - John Hands and Herb Martyr CGs | Manual ID of stomach contents | Watters 2008, Herp. Review |  |
| Sceloporus virgatus | adult | Arthropoda | Hexapoda | Insecta | Diptera |  | Chiricahua Mtns, AZ - John Hands and Herb Martyr CGs | Feeding observation | Watters 2008, Herp. Review |  |
| Sceloporus virgatus | adult | Arthropoda | Hexapoda | Insecta | Orthoptera |  | Chiricahua Mtns, AZ - John Hands and Herb Martyr CGs | Feeding observation | Watters 2008, Herp. Review |  |
| Sceloporus jarrovii | adult | Arthropoda | Chelicerata | Arachnida |  |  | Chiricahua Mtns, AZ - John Hands and Herb Martyr CGs | Manual ID of stomach contents | Watters 2008, Herp. Review |  |
| Sceloporus jarrovii | adult | Arthropoda |  | Chilopoda |  |  | Chiricahua Mtns, AZ - John Hands and Herb Martyr CGs | Manual ID of stomach contents | Watters 2008, Herp. Review |  |
| Sceloporus jarrovii | adult | Arthropoda | Hexapoda | Insecta | Coleoptera |  | Chiricahua Mtns, AZ - John Hands and Herb Martyr CGs | Manual ID of stomach contents | Watters 2008, Herp. Review |  |
| Sceloporus jarrovii | adult | Arthropoda | Hexapoda | Insecta | Diptera |  | Chiricahua Mtns, AZ - John Hands and Herb Martyr CGs | Feeding observation | Watters 2008, Herp. Review |  |
| Sceloporus jarrovii | adult | Arthropoda | Hexapoda | Insecta | Hymenoptera | Formicidae | Chiricahua Mtns, AZ - John Hands and Herb Martyr CGs | Manual ID of stomach contents | Watters 2008, Herp. Review |  |
| Sceloporus jarrovii | adult | Arthropoda | Hexapoda | Insecta | Isoptera |  | Chiricahua Mtns, AZ - John Hands and Herb Martyr CGs | Feeding observation | Watters 2008, Herp. Review |  |
| Sceloporus jarrovii | adult | Arthropoda | Hexapoda | Insecta | Lepidoptera | | Chiricahua Mtns, AZ - John Hands and Herb Martyr CGs | Manual ID of stomach contents | Watters 2008, Herp. Review |  |
| Sceloporus jarrovii | NA | Arthropoda | Hexapoda | Insecta | Orthoptera |  | Baboquivari Mtns, AZ - Kitt Peak | Manual ID of stomach contents | Goldberg & Bursey 1990. J. Herpetol. |  |
| Sceloporus jarrovii | NA | Arthropoda | Hexapoda | Insecta | Hemiptera |  | Baboquivari Mtns, AZ - Kitt Peak | Manual ID of stomach contents | Goldberg & Bursey 1990. J. Herpetol. |  |
| Sceloporus jarrovii | NA | Arthropoda | Hexapoda | Insecta | Homoptera |  | Baboquivari Mtns, AZ - Kitt Peak | Manual ID of stomach contents | Goldberg & Bursey 1990. J. Herpetol. |  |
| Sceloporus jarrovii | NA | Arthropoda | Hexapoda | Insecta | Coleoptera |  | Baboquivari Mtns, AZ - Kitt Peak | Manual ID of stomach contents | Goldberg & Bursey 1990. J. Herpetol. |  |
| Sceloporus jarrovii | NA | Arthropoda | Hexapoda | Insecta | Lepidoptera | | Baboquivari Mtns, AZ - Kitt Peak | Manual ID of stomach contents | Goldberg & Bursey 1990. J. Herpetol. |  |
| Sceloporus jarrovii | NA | Arthropoda | Hexapoda | Insecta | Diptera |  | Baboquivari Mtns, AZ - Kitt Peak | Manual ID of stomach contents | Goldberg & Bursey 1990. J. Herpetol. |  |
| Sceloporus jarrovii | NA | Arthropoda | Hexapoda | Insecta | Hymenoptera (ants) | | Baboquivari Mtns, AZ - Kitt Peak | Manual ID of stomach contents | Goldberg & Bursey 1990. J. Herpetol. |  |
| Sceloporus jarrovii | NA | Arthropoda | Hexapoda | Insecta | Hymenoptera (other) | | Baboquivari Mtns, AZ - Kitt Peak | Manual ID of stomach contents | Goldberg & Bursey 1990. J. Herpetol. |  |
| Sceloporus jarrovii | NA | Arthropoda | Chelicerata | Arachnida |  |  | Baboquivari Mtns, AZ - Kitt Peak | Manual ID of stomach contents | Goldberg & Bursey 1990. J. Herpetol. |  |
| Sceloporus jarrovii | NA | Arthropoda |  | Diplopoda |  |  | Baboquivari Mtns, AZ - Kitt Peak | Manual ID of stomach contents | Goldberg & Bursey 1990. J. Herpetol. |  |
| Sceloporus slevini | NA | Arthropoda | Hexapoda | Insecta | Homoptera |  | Southeastern AZ | NA | Newlin 1974 Angelo State University, Unpub. Dissertation |  |
| Sceloporus slevini | NA | Arthropoda | Hexapoda | Insecta | Hemiptera |  | Southeastern AZ | NA | Newlin 1974 Angelo State University, Unpub. Dissertation | |
| Sceloporus slevini | NA | Arthropoda | Hexapoda | Insecta | Hymenoptera (ants) | | Southeastern AZ | NA | Newlin 1974 Angelo State University, Unpub. Dissertation | |
| Sceloporus slevini | NA | Arthropoda | Hexapoda | Insecta | Coleoptera |  | Durango, Mexico - La Michiliá | Manual ID of stomach contents | Barbault et al. 1985, Oecologia |  |
| Sceloporus slevini | NA | Arthropoda | Hexapoda | Insecta | Orthoptera |  | Durango, Mexico - La Michiliá | Manual ID of stomach contents | Barbault et al. 1985, Oecologia |  |
| Sceloporus slevini | NA | Arthropoda | Hexapoda | Insecta | Lepidoptera | | Durango, Mexico - La Michiliá | Manual ID of stomach contents | Barbault et al. 1985, Oecologia |  |
| Sceloporus slevini | NA | Arthropoda | Hexapoda | Insecta | Hymenoptera | | Durango, Mexico - La Michiliá | Manual ID of stomach contents | Barbault et al. 1985, Oecologia |  |
| Sceloporus slevini | NA | Arthropoda | Hexapoda | Insecta | Hymenoptera (ants) | | Durango, Mexico - La Michiliá | Manual ID of stomach contents | Barbault et al. 1985, Oecologia |  |
| Sceloporus slevini | NA | Arthropoda | Hexapoda | Insecta | Hemiptera |  | Durango, Mexico - La Michiliá | Manual ID of stomach contents | Barbault et al. 1985, Oecologia |  |
| Sceloporus slevini | NA | Arthropoda | Hexapoda | Insecta | Diptera |  | Durango, Mexico - La Michiliá | Manual ID of stomach contents | Barbault et al. 1985, Oecologia |  |
| Sceloporus slevini | NA | Arthropoda | Chelicerata | Arachnida |  | Araneida | Durango, Mexico - La Michiliá | Manual ID of stomach contents | Barbault et al. 1985, Oecologia |  |
| Sceloporus slevini | NA | Plants |  |  |  |  | Durango, Mexico - La Michiliá | Manual ID of stomach contents | Barbault et al. 1985, Oecologia |  |
| Sceloporus slevini | NA | Arthropoda |  | Chilopoda |  |  | Durango, Mexico - La Michiliá | Manual ID of stomach contents | Barbault et al. 1985, Oecologia |  |
| Sceloporus jarrovii | NA | Arthropoda | Hexapoda | Insecta | Coleoptera |  | Durango, Mexico - La Michiliá | Manual ID of stomach contents | Barbault et al. 1985, Oecologia |  |
| Sceloporus jarrovii | NA | Arthropoda | Hexapoda | Insecta | Orthoptera |  | Durango, Mexico - La Michiliá | Manual ID of stomach contents | Barbault et al. 1985, Oecologia |  |
| Sceloporus jarrovii | NA | Arthropoda | Hexapoda | Insecta | Lepidoptera | | Durango, Mexico - La Michiliá | Manual ID of stomach contents | Barbault et al. 1985, Oecologia |  |
| Sceloporus jarrovii | NA | Arthropoda | Hexapoda | Insecta | Hymenoptera | | Durango, Mexico - La Michiliá | Manual ID of stomach contents | Barbault et al. 1985, Oecologia |  |
| Sceloporus jarrovii | NA | Arthropoda | Hexapoda | Insecta | Hymenoptera (ants) | | Durango, Mexico - La Michiliá | Manual ID of stomach contents | Barbault et al. 1985, Oecologia |  |
| Sceloporus jarrovii | NA | Arthropoda | Hexapoda | Insecta | Hemiptera |  | Durango, Mexico - La Michiliá | Manual ID of stomach contents | Barbault et al. 1985, Oecologia |  |
| Sceloporus jarrovii | NA | Arthropoda | Hexapoda | Insecta | Diptera |  | Durango, Mexico - La Michiliá | Manual ID of stomach contents | Barbault et al. 1985, Oecologia |  |
| Sceloporus jarrovii | NA | Arthropoda | Chelicerata | Arachnida |  | Araneida | Durango, Mexico - La Michiliá | Manual ID of stomach contents | Barbault et al. 1985, Oecologia |  |
| Sceloporus jarrovii | NA | Plants |  |  |  |  | Durango, Mexico - La Michiliá | Manual ID of stomach contents | Barbault et al. 1985, Oecologia |  |
| Sceloporus jarrovii | adult | Arthropoda | Hexapoda | Insecta | Coleoptera |  | Durango, Mexico - Las Piedras Encimadas | Manual ID of stomach contents | Gadsden et al. 2011, Southwestern Naturalist | |
| Sceloporus jarrovii | adult | Arthropoda | Hexapoda | Insecta | Hemiptera |  | Durango, Mexico - Las Piedras Encimadas | Manual ID of stomach contents | Gadsden et al. 2011, Southwestern Naturalist | |
| Sceloporus jarrovii | adult | Arthropoda | Hexapoda | Insecta | Homoptera |  | Durango, Mexico - Las Piedras Encimadas | Manual ID of stomach contents | Gadsden et al. 2011, Southwestern Naturalist | |
| Sceloporus jarrovii | adult | Arthropoda |  | Isoptera |  |  | Durango, Mexico - Las Piedras Encimadas | Manual ID of stomach contents | Gadsden et al. 2011, Southwestern Naturalist | |
| Sceloporus jarrovii | adult | Arthropoda | Hexapoda | Insecta | Hymenoptera | Formicidae | Durango, Mexico - Las Piedras Encimadas | Manual ID of stomach contents | Gadsden et al. 2011, Southwestern Naturalist | |
| Sceloporus jarrovii | adult | Arthropoda | Hexapoda | Insecta | Diptera |  | Durango, Mexico - Las Piedras Encimadas | Manual ID of stomach contents | Gadsden et al. 2011, Southwestern Naturalist | |
| Sceloporus jarrovii | adult | Arthropoda | Hexapoda | Insecta | Orthoptera |  |  | Manual ID of stomach contents | Gadsden et al. 2011, Southwestern Naturalist | |
| Sceloporus jarrovii | adult | Arthropoda | Chelicerata | Arachnida |  | Araneae | Durango, Mexico - Las Piedras Encimadas | Manual ID of stomach contents | Gadsden et al. 2011, Southwestern Naturalist | |
| Sceloporus jarrovii | NA | Arthropoda | Hexapoda | Insecta | Coleoptera |  | Chiricahua Mtns, AZ - 7mi SW Portal | Manual ID of stomach contents | Ballinger & Ballinger, 1979, Southwestern Naturalist | |
| Sceloporus jarrovii | NA | Arthropoda | Hexapoda | Insecta | Hemiptera |  | Chiricahua Mtns, AZ - 7mi SW Portal | Manual ID of stomach contents | Ballinger & Ballinger, 1979, Southwestern Naturalist | |
| Sceloporus jarrovii | NA | Arthropoda | Hexapoda | Insecta | Diptera |  | Chiricahua Mtns, AZ - 7mi SW Portal | Manual ID of stomach contents | Ballinger & Ballinger, 1979, Southwestern Naturalist | |
| Sceloporus jarrovii | NA | Arthropoda | Hexapoda | Insecta | Lepidoptera | | Chiricahua Mtns, AZ - 7mi SW Portal | Manual ID of stomach contents | Ballinger & Ballinger, 1979, Southwestern Naturalist | |
| Sceloporus jarrovii | NA | Arthropoda | Hexapoda | Insecta | Hymenoptera | | Chiricahua Mtns, AZ - 7mi SW Portal | Manual ID of stomach contents | Ballinger & Ballinger, 1979, Southwestern Naturalist | |
| Sceloporus jarrovii | NA | Arthropoda |  | Chelicerata | Arachnida |  | Chiricahua Mtns, AZ - 7mi SW Portal | Manual ID of stomach contents | Ballinger & Ballinger, 1979, Southwestern Naturalist | |
| Sceloporus jarrovii | NA | Arthropoda |  | Chilopoda |  |  | Chiricahua Mtns, AZ - 7mi SW Portal | Manual ID of stomach contents | Ballinger & Ballinger, 1979, Southwestern Naturalist | |
| Sceloporus jarrovii | NA | Arthropoda | Hexapoda | Insecta | Coleoptera |  | Chiricahua Mtns, AZ - Barfoot and Rustler Parks | Manual ID of stomach contents | Ballinger & Ballinger, 1979, Southwestern Naturalist | |
| Sceloporus jarrovii | NA | Arthropoda | Hexapoda | Insecta | Homoptera |  | Chiricahua Mtns, AZ - Barfoot and Rustler Parks | Manual ID of stomach contents | Ballinger & Ballinger, 1979, Southwestern Naturalist | |
| Sceloporus jarrovii | NA | Arthropoda | Hexapoda | Insecta | Hemiptera |  | Chiricahua Mtns, AZ - Barfoot and Rustler Parks | Manual ID of stomach contents | Ballinger & Ballinger, 1979, Southwestern Naturalist | |
| Sceloporus jarrovii | NA | Arthropoda | Hexapoda | Insecta | Diptera |  | Chiricahua Mtns, AZ - Barfoot and Rustler Parks | Manual ID of stomach contents | Ballinger & Ballinger, 1979, Southwestern Naturalist | |
| Sceloporus jarrovii | NA | Arthropoda | Hexapoda | Insecta | Lepidoptera | | Chiricahua Mtns, AZ - Barfoot and Rustler Parks | Manual ID of stomach contents | Ballinger & Ballinger, 1979, Southwestern Naturalist | |
| Sceloporus jarrovii | NA | Arthropoda | Hexapoda | Insecta | Orthoptera |  | Chiricahua Mtns, AZ - Barfoot and Rustler Parks | Manual ID of stomach contents | Ballinger & Ballinger, 1979, Southwestern Naturalist | |
| Sceloporus jarrovii | NA | Arthropoda | Hexapoda | Insecta | Hymenoptera | | Chiricahua Mtns, AZ - Barfoot and Rustler Parks | Manual ID of stomach contents | Ballinger & Ballinger, 1979, Southwestern Naturalist | |
| Sceloporus jarrovii | NA | Arthropoda | Chelicerata | Arachnida |  |  | Chiricahua Mtns, AZ - Barfoot and Rustler Parks | Manual ID of stomach contents | Ballinger & Ballinger, 1979, Southwestern Naturalist | |
| Sceloporus jarrovii | NA | Arthropoda | Chelicerata | Arachnida; Solfugae | |  | Chiricahua Mtns, AZ - Barfoot and Rustler Parks | Manual ID of stomach contents | Ballinger & Ballinger, 1979, Southwestern Naturalist | |

Appendix 2: Existing diet records collated from a literature search. We considered diet records of free-ranging adult lizards of the three species (*S. jarrovii*, *S. slevini*, or *S. virgatus*); studies in which lizards were fed or had their diets supplemented were not considered.
